# Microorganisms in the Treatment of Cancer: Advantages and Limitations

**DOI:** 10.1155/2018/2397808

**Published:** 2018-02-27

**Authors:** Klaudia Łukasiewicz, Marek Fol

**Affiliations:** Division of Cellular Immunology, Department of Immunology and Infectious Biology, Faculty of Biology and Environmental Protection, University of Lodz, Łódź, Poland

## Abstract

Cancer remains one of the major challenges of the 21st century. The increasing numbers of cases are not accompanied by adequate progress in therapy. The standard methods of treatment often do not lead to the expected effects. Therefore, it is extremely important to find new, more effective treatments. One of the most promising research directions is immunotherapy, including the use of specific types of microorganisms. This type of treatment is expected to stimulate the immune system for the selective elimination of cancer cells. The research results seem to be promising and show the intensive activation of the immune response as a result of bacterial stimulation. In addition, it is possible to use microorganisms in many different ways, based on their specific properties, that is, toxin production, anaerobic lifestyle, or binding substances that can be delivered to a specific location (vectors). This paper provides an overview of selected microorganisms which are already in use or that are in the experimental phase. Just like any other therapy, the use of microbes for cancer treatment also has some disadvantages. Nevertheless, this kind of treatment can supplement conventional anticancer therapy, giving cancer patients a chance and hope of recovery.

## 1. Introduction

According to the report of Ferlay et al. [1], it is estimated that in 2012 in Europe, approximately 3.45 million people suffered from different types of cancer and 1.75 million died. Cancers of breast, rectum or colon, lung, and prostate are responsible for half of all cancer cases in Europe; furthermore, the first three of them and additionally the stomach cancer are the most common causes of death from cancer in the European Union. Cancer is the second major cause of death in the USA. It is prognosticated that during the year 2017, more than 1.6 million cases will be registered, which means that more than 4600 cancer cases will be reported every day [2]. It should be stressed that the statistics may be underestimated as many cancer lesions develop over the years and are only diagnosed at a high stage of the disease. There are many factors that influence the development of cancer. One of the best recognized risk factors is tobacco smoking, which can cause cancers in lungs, head, and neck [3]. Other examples are chemicals, including those being in use in the research laboratories, such as ethidium bromide, which is a highly mutagenic agent [4]. Mutations in the genetic material may also be the result of irradiation, such as UV or X rays [5], or the effect of infection with a pathogen such as HPV (cervical cancer) [6] or HCV and HBV (liver cancer) [7, 8]. Neoplasms can also be inherited as a polygenic disorder. This is due to the overlap of hereditary changes in the carriers of the defective gene and the DNA damage at sites that are important for the process of cancerogenesis that occurred during human development. The flagship examples are inherited damaged BRCA 1, which is responsible for the development of breast and ovarian cancer, and RB1, which is responsible for the development of retinoblastoma [9]. There are some genetic predispositions, so-called “genetic background,” including for instance single mutations in the genetic material or epigenetic changes that may increase the risk of cancer development [10].

The main priorities in cancer research are prevention, early detection, and the development of new therapies, including personalized therapies, which are intended to include the molecular biology of a particular tumor and the predisposition of the patient's immune system. Among the known and practiced anticancer therapies, the use of microbes appears to be one of the most original strategies. Although now somewhat forgotten, it has a large potential to play a significant role in the treatment of cancer. This paper reviews the perspectives for the use of microorganisms in anticancer therapy. It presents microorganisms that have already been commonly used and those going through phase II and phase III clinical trials.

## 2. Anticancer Therapy: General Characteristics

Treatment methods can be divided into local/regional treatment and systemic treatment. The combination of both methods is combination therapy. Local treatment includes oncological surgery and radiotherapy. Surgical treatment plays the most important role in cancer treatment, because it often gives a chance of a complete cure (radical treatment). In addition, it is used in palliative treatment that does not give any chance of a cure, but allows incurable patients to alleviate the symptoms of the disease and ensure optimal functioning in the last months of life. Surgery also allows reducing the tumor mass, which significantly improves the effects of systemic treatment. Another method of regional treatment is radiotherapy, which involves irradiation of the tumor, leading to impaired cell division capacity and metabolic functions. Radiotherapy can be applied in two ways: using an external source and by introducing a source into or near a tumor [11]. Systemic treatment has a smaller or greater impact on the entire body of the patient. We can distinguish between chemotherapy, hormone therapy, and biological therapy. Chemotherapy uses drugs that block cell division. All quickly divided cells are destroyed—both cancerous and normal cells of the body. Chemotherapy is accompanied by a number of side effects and general worsening of the patient's condition. Therefore, new, more precise treatments are being sought [11]. Hormone therapy is used in tumors that express receptors for appropriate hormones, such as breast, prostate, or ovary. This method is based on hormonal imbalance, but it is important to evaluate the expression of receptors before treatment, as they may change with the progression of the tumor. Hormone therapy is primarily used for tumor recurrences [12]. In terms of biological treatment, therapy with monoclonal antibodies plays a dominant role. They are directed against specific antigens of tumor cells. In addition, biological substances are used that block the pathways of cancer cell metabolism. Anticancer therapy also involves vaccination with the use of precisely prepared dendritic cells or cancer cells. Interestingly, as early as decades ago, the immunization with the use of microorganisms was already applied as anticancer therapy to stimulate the patient's immune system to fight the disease; however, this kind of treatment is currently poorly explored [13, 14].

### 2.1. Microorganisms as an Element of Immunotherapy

Intrusion of microorganisms into the body leads to the activation of immune mechanisms, which manifests itself in increasing the number and recruitment of congenital immune cells (especially neutrophils, monocytes/macrophages, and NK cells), activation of acquired immunity cells, that is, T and B lymphocytes, and intensification of proinflammatory cytokine production. It is assumed that the “mobilized” immune system, by intentionally introducing microorganisms into the oncological patient, is able to at least limit the development of cancer. This is a method in which microbes indirectly lead to cancer regression—especially in those in whom other commonly used treatments have failed [15]. The safety of the used microorganisms is extremely important, because the aim of the therapy is to fight cancer, not to harm the patient's organism by infecting it with a pathogen. Various methods are used to ensure the safety of the formulations [16]. First and foremost, microbes are deprived of their pathogenicity (attenuation), for example, by culturing under appropriate environmental conditions or by the treatment of certain substances, resulting in mutation and weakening/loss of pathogenic properties [17].

Bacteria can be applied in various forms for therapeutic purposes. Apart from whole, living attenuated cells, we can use genetically engineered bacteria expressing particularly desirable factors [18]. Microorganisms are also applied as vectors, which are carriers of specific antineoplastic agents (e.g., chemotherapeutics) or enzymes useful in cancer cell destruction. The use of bacteria as a vector to transfer a chemotherapeutic agent directly into the tumor allows a significant reduction of the side effects of treatment that usually accompany traditional chemotherapy [18, 19]. In addition, there is a therapeutic potential in using bacterial secretion products, for example, toxins. Their presence in the tumor environment could have destructive effect on cancer cells [18, 20–22]. The use of sporangial bacteria, which can survive under unfavorable environmental conditions, represents another approach, which has been applied in the experiments with *Clostridium novyi*. This microorganism prefers anaerobic conditions, which are found in the tumor. Instead of spreading over the entire organism, the bacteria are directed to the tumor site only, where they have the optimal conditions for growth. This bacterial property allows the patient to be protected against the development of serious infections [16].

## 3. Back to Sources

The beginnings of the use of microbes in cancer therapy date back to the nineteenth century. Dr. William Coley (1862–1936) developed a mixture of bacterial microbes and, for the first time in modern medicine, he successfully treated certain types of cancer, thus becoming the father of immunotherapy [23]. Dr. William Coley was employed at the New York Cancer Hospital and then at the Hospital for Special Surgery in New York, as a surgeon specializing in sarcoma, especially bone cancer. He was deeply shocked when one of his first oncological patients died, and that was a reason he began seeking more effective forms of cancer treatment. Coley studied in-depth the case report forms of his contemporary and much earlier oncological patients. He came across information on spontaneous regression of sarcoma in patients with severe bacterial infection. This prompted him to undertake experimental therapy, which involved the administration of *Streptococcus pyogenes* to a patient with nonoperative bone sarcoma. The results were extremely promising because remarkable tumor regression was observed [24]. Coley worked on new treatment method for the next forty years, preparing other variants of microbial mixture. This preparation could be called a vaccine because it stimulated/activated the immune system by the introduction of antigens (bacterial components). Twenty different versions of the vaccine (called the Coley's toxin) were devised at that time, and each of them had a different effectiveness [25]. It is also very important that the microbes were administered in different ways: intramuscularly, intravenously, or directly into the tumor. Coley's toxin was given to hundreds of patients, and more than a quarter of them were cured. After Coley's death, due to the lack of systematic and precise documentation on the research methodology and preparation of the vaccines, such spectacular results were not replicated [24]. Nevertheless, many attempts were undertaken to reconstitute Coley's toxin [23], for example, by the company MBVax. It has been decided to reproduce the version of a vaccine that was created by the bacteriologist Martha Tracy, who co-operated with William Coley at that time. This vaccine was supposed to have the greatest efficacy. The formulation was based on two types of microorganisms: beta-hemolytic *Streptococcus pyogenes*—as the main factor activating the immune system, and *Serratia marcescens*, producing a red colorant, prodigiosin, which is an apoptosis factor of tumor cells [25, 26]. After administration of the bacteria to the patient, there was a significant increase in the level of cytokines as well as the number of neutrophils, macrophages, T and B lymphocytes, and NK cells. The antigen-presenting cells (APC) initiated the immune response by presenting bacterial antigens to naive CD4^+^ T cells and CD8^+^ cells, leading to the production of proinflammatory cytokines, such as interleukins (IL) 1, IL-2, and IL-12, and tumor necrosis factor alpha (TNF), but a total regression of the tumor occurred only in one case [25, 27].

Currently, Coley's toxin is not used in the treatment of bone sarcoma, but the so-called antineoplastic vaccines are commonly used in the treatment of other cancers. The method based on stimulating the immune system is constantly being developed, and more and more studies on immunotherapy appear. Attention is particularly focused on finding new ways to induce the production of proinflammatory cytokines, including TNF or interferon (IFN), which have the capacity to destroy tumor cells. That is why it can be claimed that Dr. William Coley was ahead of his time [24].

## 4. Bacteria Used as Anticancer Agents

The antitumor efficacy of microorganisms is extremely diverse. Results of clinical trials allow determining whether a particular product can be intended for general use. Currently used anticancer bacterial microbial preparations have the status of a therapy complementary to standard treatment, increasing the patient's chances of complete recovery. The chapter reviews the microorganisms going through phase II and phase III clinical trials and presents those that have already been commonly used in cancer therapy.

### 4.1. *Mycobacterium bovis* BCG

Bacillus Calmette-Guérin (BCG) is a strain of *Mycobacterium bovis* developed by Albert Calmett and Camille Guérin as a tuberculosis vaccine and has been used since 1921. In many countries, this vaccine has been induced in the mandatory vaccination schedule and is administered to children within 24 hours after birth, in a single dose, intradermally.

*Mycobacterium bovis* is an etiological agent of bovine tuberculosis. However, in certain circumstances (e.g., after ingestion of untreated milk from an infected animal), it can cause tuberculosis symptoms in humans as well. That is why it was necessary to attenuate this microorganism. Calmett and Guérin have passaged *M. bovis* (231 passages in total) for 13 years on a medium consisting mainly of cooked potato slices soaked in ox bile and glycerin. Only then did it become safe for human use, as an avirulent but immunogenic strain [28].

At the beginning of the twentieth century there were some links between the occurrence of tuberculosis and cancer regression [28]. However, only after Morales and his colleagues demonstrated in 1976 that the use of BCG was accompanied with the cancer regression, the vaccine was approved as the complementary treatment of bladder cancer [29]. Treatment of this type of cancer with the *M. bovis* BCG strain requires the intravesical infusion of the microbial suspension using urethral catheters. This therapy is most often used after resection to eliminate accurately the cancer cells and to prevent recurrence [29]. The dose and duration of treatment are strictly dependent on the stage of cancer. Clinical observations show that recurrence is much less likely to occur after tumor resection or resection and chemotherapy when BCG is administered intravesically [30].

BCG's mechanism of action is based on stimulating the patient's immune system. It appears that IFN-*γ* and effector cells, that is, CD4^+^ and CD8^+^ lymphocytes, play an extremely important role in the recognition of tumor antigens. In addition, the pool of proinflammatory cytokines is increasing, which enhances the immune response of the body by activating the phagocytosis of cancer cells. Providing the selected vitamins during therapy may increase the survival of *M. bovis* BCG cells, which improves the quality of therapy [16, 29, 31, 32].

### 4.2. *Streptococcus pyogenes* OK-432

*Streptococcus pyogenes* was originally used in the treatment of bone sarcoma by Dr. William Coley. However, the emergence and development of other treatments for cancer, especially chemotherapy and radiotherapy, caused that for many years, the concept of using this microorganism was forgotten. Fortunately, the concept of anticancer therapy with the use of *S. pyogenes* has endured and the bacteria are currently applied in the treatment of lymphangiomas in children. Presently, the *S. pyogenes* OK-432 strain has been used in that way in many countries around the world [25, 33].

Lymphangiomas are tumors formed by excessive division of lymphatic vessels' endothelial cells. They are most often found in the head and neck area of children under the age of two. The pathological development of lymphatic vessels is primarily associated with impaired lymph flow, which in turn manifests itself in the formation of cysts. Changes in children resemble goiter, similar to that one, which is associated with an enlarged thyroid gland. Treatment primarily involves surgical removal of the cyst, but this is not an easy task, and is often burdened with numerous adverse effects, including death [34, 35].

An alternative and safer method of treatment is sclerotherapy. *Streptococcus pyogenes* OK-432 is injected into pathologically changed lymphatic vessels. In Japan, this microorganism has been successfully used in the treatment of lymphangiomas in children since 1987. Studies show that the strain is safe and results in at least 50% reduction of cyst volume [33, 35].

The mechanism of action of the microorganism is also based on the sensitization of the immune system. Activated cells destroy the neoplasm, further growth is inhibited, and the lymphangioma is reduced. Studies using flow cytometry have shown that the first day after suspension administration, the numbers of neutrophils and macrophages, as well as lymphocytes, rapidly increase. NK CD56^+^ cells, TNF*α*, IL-6, IL-8, IFN*γ*, and VEGF (vascular endothelial growth factor) levels also increase. Due to the appearance of inflammation immediately after the procedure, the lesion may be swollen, but therapeutic effects are noticeable after a few months [33, 35–37]. Moreover, studies conducted in the years 2005–2015 showed the great effectiveness of this strain also in the treatment of intraoral ranula. Complete regression occurred in 78.2% of patients [38].

### 4.3. *Clostridium novyi* and *Salmonella enterica* Serovar Typhimurium

Obligate anaerobes and facultative anaerobes have potential to be used in anticancer therapies because they grow best under conditions of significant oxygen unavailability (hypoxia). Oxygen is delivered to the cells through blood vessels which penetrate mainly the tumor surface area. That results in impaired diffusion of oxygen into the tumor and hypoxia. The anaerobic environment creates favourable conditions for the development of anaerobic bacteria, for example, *Clostridium* spp., *Salmonella spp*., *Bifidobacterium* spp., or *Listeria spp*. [16, 39]. The greatest advantage of using these microorganisms is that they locate directly inside the tumor, in contrast to chemotherapeutics, which spread throughout the body with blood, also destroying normal, healthy cells [39–41].

In the context of hypoxia and the antineoplastic therapy, the most common type of bacteria being in use is *Clostridium*, due to the anaerobic nature of the rods. Bacteria develop in the tumor's necrotic areas and can directly damage tumor cells [39–41]. The history of the use of *Clostridium* in the fight against cancer dates back to 1935, when Connell published an article describing the regression of advanced cancer under the influence of enzymes produced by *Clostridium histolyticum* [42]. Since then, more research has been done on the use of *Clostridium*. The attenuated strain of *Clostridium novyi*-NT has positively undergone phase I and phase II clinical trials, giving extremely promising results for the treatment of leiomyoma [39–41]. The mechanism of the anticancer activity of *Clostridium* spp. is unknown yet, but it is common knowledge that bacterium is capable of producing specific enzymes and toxins that destroy cancer cells. In addition, it produces specific proteins that can be conjugated to specific chemotherapeutics. This allows the drug to enter the tumor. In traditional chemotherapy, drugs are not able to penetrate into the tumor precisely due to its external vascularization and internal hypoxia [39–41].

*Salmonella enterica* serovar Typhimurium, an etiological agent of typhoid fever, shows similar features as *Clostridium*. It is a relatively anaerobic rod that can also be located in the necrotic tumor regions. In the treatment of cancer, the attenuated strain *Salmonella typhimurium* VNP20009 is used for safety reasons [43]. Clinical trials on the use of this microorganism for melanoma treatment began in 2002 [16]. In addition, the VXM01 antitumor vaccine, which is based on the attenuated strain of *Salmonella typhi*, has successfully passed phase I clinical trials. This bacterium has a plasmid-encoding expression of VEGFR2 (vascular endothelial growth factor receptor-2). The vaccine blocks the angiogenesis process. The formulation was tested in individuals with pancreatic cancer [44].

## 5. Perspectives for the Use of New Species of Microorganisms

Man has always looked for a mythical panacea, the cure for every illness. Alchemists sought it out in the Middle Ages. Such a legendary substance does not exist, but ideal drugs are still sought by biologists, chemists, physicians, and the other researchers. Ideal means as much as possible safe and effective. This concept is also rooted in research into cancer therapies, which can be evidenced by ever more courageous and original ideas, including the use of microbes; today too daring, in the future they could set standards [45].

### 5.1. *Magnetococcus marinus*

The most recent anticancer strategies use the achievements of various scientific disciplines, for instance, nanobiotechnology. Nanoparticles (nanocapsules), lipid vesicles with a chemotherapeutic drug inside, are the object of growing interest. Nanoliposomes are able to deliver the drug inside the tumor [46]. However, they are not a perfect solution because many of the particles do not reach the target. As mentioned earlier, the tumor is only vascularized from the outside, which makes it impossible for chemotherapeutics to reach the inside of the lesion. Hence, the idea of delivering drugs directly to the tumor with vectors/carriers would allow for more precise targeting of the cancer site. Limiting the spread of the drug only to the tumor area would significantly reduce the adverse effects of chemotherapy [40]. For the mentioned reasons, it was decided to take a closer look at very original bacteria named *Magnetococcus marinus* MC1 [19].

*Magnetococcus marinus* MC1 is a Gram-negative coccus found in the Atlantic Ocean near Rhode Island, USA. This microorganism has cilia, arranged in two bundles located at one pole, which enable the bacteria to move. The unique feature of this bacterium structure is the presence of magnetosomes—special elements which are magnetite particles (Fe_3_O_4_) surrounded by membranes, forming chains in the cytosol [47]. The presence of magnetite orients the bacteria with the Earth's magnetic field. In addition, this microorganism shows negative aerotaxis capacity, that is, prefers an environment that is poor in oxygen [48]. These properties make the *Magnetococcus marinus* a useful tool to destroy cancer cells. Using a powerful magnetic field, the same as in the MRI technique (magnetic resonance imaging), it would be possible to direct bacteria containing magnetosomes to the site of the tumor. The bacteria will be located precisely in the areas of hypoxia, in that case inside the tumor, where they would deliver a chemotherapeutic encapsulated in nanoliposomes attached to the bacteria surface. Animal studies have shown that approximately 55% of nanoliposomal transmission cells reach tumors [19, 48].

### 5.2. *Toxoplasma gondii*

*Toxoplasma gondii* is an obligatory intracellular protozoan. It can be life-threatening to people with impaired immunity or pregnant women, who can suffer abortion or foetal malformation. The primary hosts are *Felidae* (e.g. cats), in which the sexual phase of pathogen development occurs. Feces containing parasite's oocytes are the source of infection for birds and mammals, including rodents and people, who are the intermediate hosts. In the intermediate hosts' organisms, the parasite divides and cysts are formed in the muscles and brain. In healthy individuals, the immune system inhibits further development of the protozoa [49, 50].

It turns out that the protozoan and its lysate, called TLA (Toxoplasma lysate antigen), containing antigens of the microorganism, can be used to treat not only neurodegenerative diseases [49] but also cancer [49, 51, 52]. In particular, the research focuses on the use of the uracil auxotrophic carbamoyl phosphate synthase mutant *Toxoplasma gondii* (CPS) in the treatment of the most aggressive types of cancer: melanoma, pancreatic cancer [53], lung cancer [49, 52], and ovarian cancer [54]. As a result of the administration of this strain, an increase in the level of IL-12, a cytokine which mediates the inflammation, and the activation of other immune cells were observed. In addition, IL-12 is responsible for inhibition of angiogenesis, leading to hypoxia and tumor growth slackening [54]. Moreover, the expression of the CD31 molecule (angiogenesis marker) is reduced, and the Th1 lymphocytes appear, which also causes a significant inhibition of the formation of blood vessels [49]. In addition, CD4^+^, CD8^+^, and IFN*γ* levels are significantly increased [53]. Recent studies in the mouse model indicate that the use of *T*. *gondii* CPS therapy provides long-term protection from recurrence, which is connected with the development of immune memory and the high titre of IgG recognizing the specific tumor's antigens [53].

### 5.3. *Plasmodium falciparum*

Malaria, caused by protozoa of the genus *Plasmodium*, is one of the most common parasitic diseases in the world. The parasite is transmitted from a healthy person through an *Anopheles* mosquito. The life cycle includes two hosts, an intermediate host—a human being, and a primary one—a mosquito. When the mosquito bites, sporozoites enter the body through blood vessels and then move to the liver where they enter hepatocytes very rapidly, thanks to the apical complex, and in that way, they avoid contact with the host immune system. Here, the sporozoites form schizonts, within which there are numerous divisions, and merozoites are formed. Merozoites are released into the bloodstream about 30 days after the infection. From this point, an erythrocytic cycle starts, and it is responsible for the clinical symptoms of malaria. Merozoites penetrate erythrocytes and turn into trophozoites and then again into schizonts with merozoites inside. Every 48 hours, new merozoites are released and the cycle repeats, destroying more and more red blood cells. After several cycles, some of the merozoites create gametocytes that can be sucked out with blood by a mosquito. There is sporogenesis (a sexual development phase) inside the mosquito's digestive system. Gametes in the body of the mosquito combine to create a zygote and then an ookinete that penetrates the intestinal epithelium of the mosquito, forming an oocyte [55, 56].

*Plasmodium falciparum* is considered to be the most malignant causative agent of malaria because it aggregates erythrocytes and thrombocytes that adhere to the vascular endothelium, which can lead to the closure of vascular light and thus damage to vascular walls and even necrosis [57]. However, despite all the negative features of the parasite, it can be used to treat cancer. Salanti et al. [22] demonstrated that *Plasmodium falciparum*, after penetrating into erythrocytes, expresses malarial protein VAR2CSA, which is responsible for binding to mucopolysaccharide-chondroitin sulphate A (CSA), present in physiological conditions on the surface of placenta cells [22, 56, 58]. The placenta is a specialized organ whose main function is acting as a mediator between the mother and the baby. It develops extremely fast—from the time of implantation of the embryo into the uterus until the fetus' heart is a fully functional organ. Cells proliferate, and proangiogenic factors cause vascularization of the placenta, which develops and grows throughout the pregnancy, forming a cellular syncytium [58]. It turns out that the placenta and tumors have more in common than just the cell proliferation rate. Chondroitin sulphate is also present on the surface of many tumor cells. Thus, the rVAR2 protein, which is a recombinant version of the VAR2CSA malarial protein, was developed and after being conjugated to the appropriate part of the diphtheria toxoid it was tested for suitability in the destruction of tumor cells. Both *in vitro* studies on tumor cell lines and *in vivo* studies on the mouse model showed the high effectiveness of the strategy used, with the best effects observed for certain types of melanoma with high expression of chondroitin sulfate [22, 58].

## 6. Summary and Conclusions

Anticancer therapy with the use of microorganisms is often marginalized and neglected. A very narrow group of researchers strive to investigate and develop cancer treatment methods using microorganisms, either as vaccines that activate the immune system to fight disease or as vectors for the transmission of antitumor therapeutics. Very often, these studies go unnoticed, despite significant achievements in the field of immunotherapy. With this method of treatment, people who have been failed by conventional treatment are more likely to recover, what is more important, this type of therapy is more selective and therefore less burdensome for the entire organism of the patient [18–22]. Of course, like every treatment, this one also has certain disadvantages. There is primarily a risk of developing infection and related consequences, including death. In experimental studies, laboratory animals have been used to show that the most effective strain actually destroyed cancer, but animals died because of infection by pathogens. It is therefore very important to ensure the safety of the patients, especially by using only adequately attenuated microorganisms. Only a perfect balance between the attenuation of a microorganism and its immune stimulatory ability can guarantee the proper effect. In addition, the costs associated with clinical trials and the introduction of a new product to the market are extremely high. Legal regulations are also very complicated, due to the not fully known impact of microbes on cancer.

Another issue is the need to take into account the patient's condition. An accurate diagnosis and carrying out proper tests are absolutely necessary. The research on *Plasmodium falciparum* is a good example of how difficult it is to move from the experimental phase to the implementation stage. Another concern at the moment is the limited use of microbial preparations. As mentioned above, there are over two hundred different cancer diseases and only for a few of them where the bacterial preparations have been developed or introduced. So far, there is no general-purpose (universal) bacterial preparation, each type of cancer requires a specially selected (optimized) strain (Table 1), and it is difficult to believe that this kind of universal microbe-based treatment could be ever compiled. However, microbial therapy and research on other bacterial preparations should not be stopped. Relatively recently, a number of reports have been published regarding the use of a padeliporfin derivative (palladium bacteriopheophorbide monolysine taurine, WST-11) in the treatment of prostate cancer [59–62]. This is a vascular-targeted photodynamic therapy (VTP) with the use of the water-soluble WST-11 complex directly administrated into the tumor and subsequently a 753 nm wavelength laser beam aiming the cancer cells to activate the compound. WST-11, in contact with infrared light, induces the synthesis of reactive oxygen species and inhibits angiogenesis, which leads to tumor necrosis. The compound that was the starting point for WST-11 was isolated from ocean-bottom bacteria. The bacteria have developed photosynthetic pigment (bacteriochlorophyll) to adapt to near-total darkness. They use the smallest light source as energy. The success of this therapy undoubtedly proofs of the need for further research into the use of microbes and their compounds/products in the treatment of cancer.

## Figures and Tables

**Table 1:** A representative list of microorganisms used/planned to be used in anticancer therapy.

Microorganism	Strain/antigen	Cancer	Type of treatment	Deployment
*Mycobacterium bovis*	Atenuated strain Calmette-Guérin	Superficial bladder cancer	Complementary therapy	Commonly used
*Streptococcus pyogenes*	OK-432	Lymphangioma	Alternative therapy for surgical treatment	Commonly used
*Clostridium novyi*	Strain NT	Solid tumors	No data	Clinical trials
*Salmonella enterica* serovar Typhimurium	Strain VNP20009	Melanoma	No data	Clinical trials
*Magnetococcus marinus*	MC1	Solid tumors and some metabolic tumors	Additional therapy supporting chemotherapy	Experimental research (animal studies)
*Toxoplasma gondii*	*CPS*/TLA	Pancreas, lung and ovarian cancer, and melanoma	No data	Experimental research
*Plasmodium falciparum*	rVAR2-DT	Melanoma expressing CS	No data	Experimental research
